# Oxidative Stress Challenge Uncovers Trichloroacetaldehyde Hydrate-Induced Mitoplasticity in Autistic and Control Lymphoblastoid Cell Lines

**DOI:** 10.1038/s41598-017-04821-3

**Published:** 2017-06-30

**Authors:** Richard Eugene Frye, Shannon Rose, Rebecca Wynne, Sirish C. Bennuri, Sarah Blossom, Kathleen M. Gilbert, Lynne Heilbrun, Raymond F. Palmer

**Affiliations:** 1 Arkansas Children’s Research Institute, Little Rock, AR USA; 2 Department of Pediatrics, University of Arkansas for Medical Sciences, Little Rock, AR USA; 3 Department of Family and Community Medicine, University of Texas Health Science Center, San Antonio, TX USA

## Abstract

Mitoplasticity occurs when mitochondria adapt to tolerate stressors. Previously we hypothesized that a subset of lymphoblastoid cell lines (LCLs) from children with autistic disorder (AD) show mitoplasticity (AD-A), presumably due to previous environmental exposures; another subset of AD LCLs demonstrated normal mitochondrial activity (AD-N). To better understand mitoplasticity in the AD-A LCLs we examined changes in mitochondrial function using the Seahorse XF96 analyzer in AD and Control LCLs after exposure to trichloroacetaldehyde hydrate (TCAH), an *in vivo* metabolite of the environmental toxicant and common environmental pollutant trichloroethylene. To better understand the role of reactive oxygen species (ROS) in mitoplasticity, TCAH exposure was followed by acute exposure to 2,3-dimethoxy-1,4-napthoquinone (DMNQ), an agent that increases ROS. TCAH exposure by itself resulted in a decline in mitochondrial respiration in all LCL groups. This effect was mitigated when TCAH was followed by acute DMNQ exposure but this varied across LCL groups. DMNQ did not affect AD-N LCLs, while it neutralized the detrimental effect of TCAH in Control LCLs and resulted in a increase in mitochondrial respiration in AD-A LCLs. These data suggest that acute increases in ROS can activate mitochondrial protective pathways and that AD-A LCLs are better able to activate these protective pathways.

## Introduction

Environmental toxicants have been proposed to play a role in the etiology of many diseases. The underlying mechanisms in which toxicants cause disease include immune activation^1–3^ and disruption of redox and mitochondrial metabolism^4–6^. Mitochondrial dysfunction can disrupt cellular physiology through many mechanisms, including epigenetic changes and modulation of cellular signal transduction pathways^5, 7–10^ and has been implicated in a wide variety of diseases including neurodevelopmental, neurological and psychiatric disorders, diabetes, cancer, and cardiovascular and kidney disease^11^. The association of mitochondrial dysfunction with these diseases may reflect the significant vulnerability of mitochondria to endogenous and exogenous stressors^12^, partially due to its complicated genetics^13^.

Toxicants can both damage the mitochondria and cause mitoplasticity. Mitoplasticity refers to the mitochondria’s ability to adapt or transform to optimally function in the face of changes in energy demand, substrate availability or physiological stress^5^. Mitoplasticity involves a wide number of pathways, including enzymes in the citric acid cycle and electron transport chain (ETC), proton leak, redox regulation, transcription factors, and mitochondrial repair and regeneration such as mitochondrial biogenesis, mitophagy and mitochondrial fission and fusion^5^.

Trichloroethylene (TCE) is an environmental toxicant that causes neurobehavioral abnormalities in animals^14, 15^ through mitochondrial^16–19^ and redox abnormalities^20, 21^, immune dysregulation^22–25^ and epigenetic changes^14^. The effect of TCE on the mitochondria appears paradoxical. TCE causes ETC dysfunction in isolated mitochondria, but also increases the expression of genes that enhance mitochondrial function. Acute exposure to TCE results in inhibition of state 4 respiration in isolated mitochondria^17^ and decreased ETC Complex I activity in chronically exposed rodents^16, 18^. However, other studies show that TCE exposure increases the expression of mitoplasticity related genes, including peroxisome proliferator-activated receptor-gamma coactivator-1 alpha (PGC1α) which, in turn, up-regulates mitochondrial metabolism genes, including nuclear respiratory factors (NRF-1, NRF-2), uncoupling proteins (UCP), ETC genes, mitochondrial gene transcription, and genes essential for redox metabolism, including superoxide dismutase, catalase, and glutathione peroxidase-1^26–29^.

TCE has been linked to human disease including Parkinson’s Disease^16, 19, 30, 31^ and Autism Spectrum Disorder (ASD)^6, 32^. ASD is a heterogeneous neurodevelopmental disorder that may affect up to 1 in 45 individuals^33^. Studies suggest that ASD is caused by environmental factors interacting with genetic susceptibility^34, 35^ and many studies have linked environmental toxicants, including TCE, with ASD^6, 36^. Interestingly, some of the same physiological abnormalities caused by TCE exposure are associated with ASD, including mitochondrial dysfunction^8, 9^, redox abnormalities^37–39^ and immune dysregulation^40^.

We developed a lymphoblastoid cell line (LCL) model of ASD designed to understand how mitochondrial function interacts with reactive oxygen species (ROS). We have repeatedly demonstrated that a subset of LCLs (called AD-A LCLs) derived from children with ASD show an elevation in Reserve Capacity, a parameter that is tightly correlated with mitochondrial and cellular health, suggesting that the LCLs had adapted, at some point, to be more resilient at baseline^41, 42^. However, this subset of LCLs also demonstrated vulnerability to ROS such that Reserve Capacity declines at lower levels of ROS as compared to ASD LCLs without the baseline elevation in Reserve Capacity (called AD-N LCLs).

We believe that studying functional responses of mitochondria to TCE exposure can provide some insight into the functional consequences of mitoplasticity and the nature of the changes in mitochondrial function found in AD-A LCLs. First, we note that mitoplasticity pathways appear to be activated in AD-A LCLs at baseline and with TCE exposure. Second, we note the paradoxical effect of TCE on mitochondrial function; TCE exposure causes dysfunction in isolated mitochondria while other studies suggesting enhancement of mitochondrial function with TCE exposure. This is paralleled in the ASD literature as some studies demonstrate a high incidence of ETC dysfunction^43, 44^ while others demonstrate highly active mitochondria at baseline^41, 42, 45^. Third, we believe it is important to study TCE exposure in the context of AD LCLs since TCE has been linked to ASD.

TCE is water insoluble and difficult to deliver to cell culture. In addition, immune cells do not have the cytochrome p450s to metabolize TCE into its active metabolites. Therefore, in this study, we used trichloroacetaldehyde hydrate (TCAH), a major oxidative metabolite of TCE that is known to have similar adverse physiological effects as TCE^22–25^.

It is our hypothesis that exposure to TCAH will result in mitoplasticity in control LCLs that functionally manifests similar to the changes seen in the AD-A LCLs at baseline. Such a finding would support the notion that AD-A LCLs may have previously undergone mitoplasticity-associated changes as a consequence of exposure to an environmental stressor. We believe that TCAH-induced mitoplasticity may require a stressor to manifest. That is, without a stressor stimulus, the cell may not up-regulate mitoplasticity genes and mitochondrial function may either be normal or reduced due to residual damage to the ETC from TCAH exposure. This would explain why ETC enzymes, examined in isolation and separated from cellular physiology, appear to function below normal. Such a context dependent change in ETC function has been reported in chronic multiple sclerosis lesions, where complex IV activity is dependent on whether or not there is active inflammation^46, 47^. We will examine this by increasing intracellular ROS similar to previous studies^41, 42^. Interestingly, AD LCLs are already under intrinsic elevation in oxidative stress, so this may be the reason some manifest mitoplasticity at baseline. Third, we will examine the differential response to TCAH in the AD subgroups. If our hypothesis is correct, the AD-A LCLs should be more resilient to TCAH than the AD-N LCLs since the AD-As have undergone mitoplasticity to be more resilient to environmental stressors.

## Results

### Effect of TCAH on Mitochondrial Respiration in Control LCLs

#### ATP-Linked Respiration

**Figure 1:**
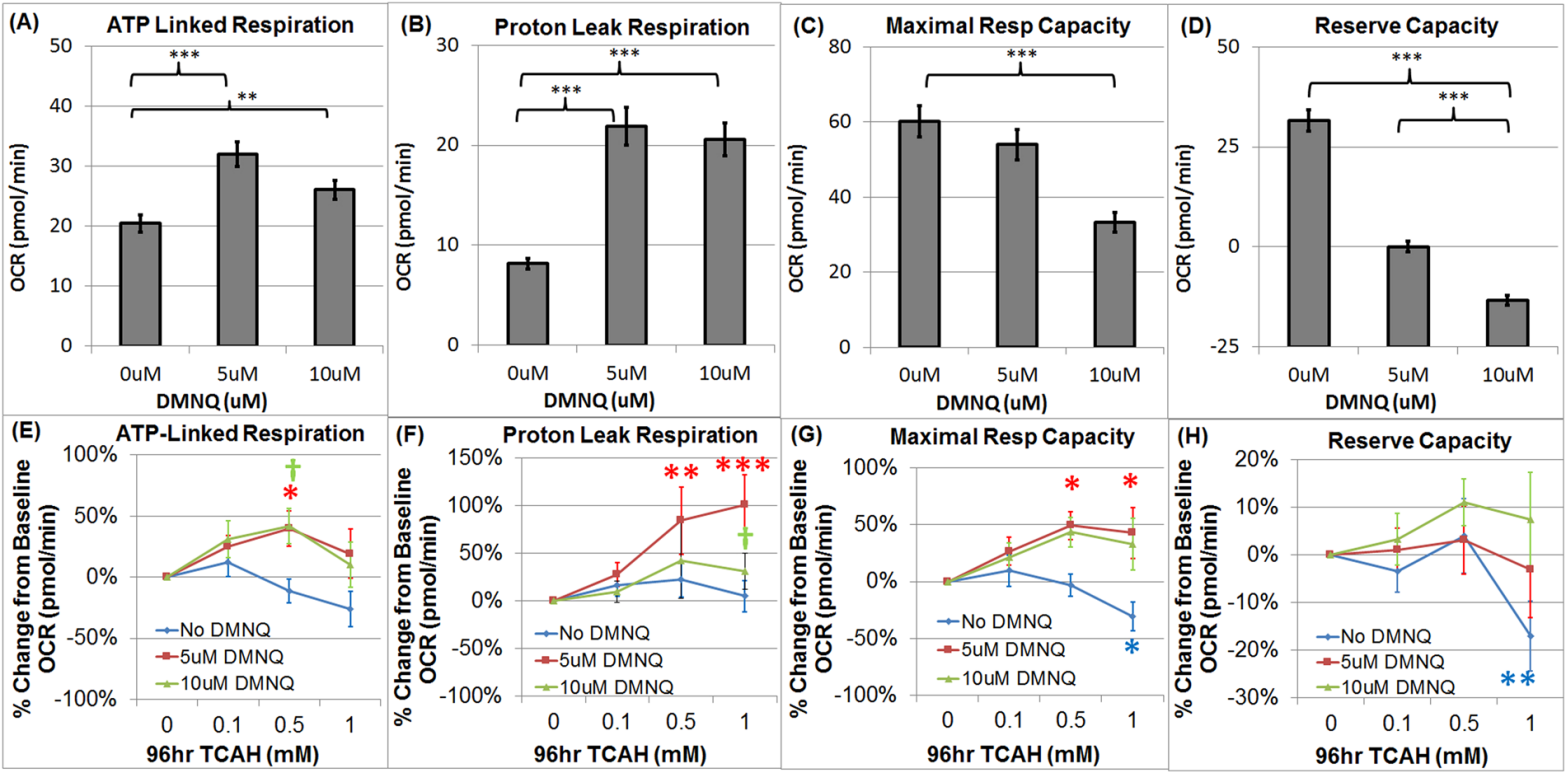
(**A**–**D**) Absolute Change in Mitochondrial Function with 2,3-dimethoxy-1,4-napthoquinone (DMNQ) in Control Lymphoblastoid cell lines (LCLs) and (**E–H**) Relative (Percent) Change from Baseline in Mitochondrial Function after exposure to Trichloroacetaldehyde hydrate (TCAH). ATP-linked Respiration (**A**) and Proton Leak Respiration (**B**) increase with exposure to DMNQ. Maximal Respiratory Capacity (**C**) and Reserve Capacity (**D**) decrease with exposure to DMNQ. Without acute DMNQ exposure, TCAH reduces Reserve Capacity but acute DMNQ exposure appears to mitigate the effect of TCAH by increasing Maximal Respiratory Capacity. (**E**) ATP-linked Respiration increased with TCAH exposure only when TCAH exposure was followed by acute exposure to DMNQ; (**F**) Proton Leak Respiration increased with TCAH exposure only when followed by acute exposure to DMNQ; (**G**) Maximal Respiratory Capacity decreases with TCAH without acute DMNQ exposure but increases with TCAH exposure when followed by acute exposure to DMNQ. (**H**) Reserve Capacity significantly decreased at the highest TCAH concentration when LCLs were not acutely exposed to DMNQ but this effect was mitigated when TCAH exposure was followed by acute DMNQ. The line color correspond to the DMNQ exposure (Blue = No DMNQ; Red = 5 uM DMNQ; Green = 10 uM DMNQ). ^†^p <  = 0.05; *p <  = 0.01; **p <  = 0.001; ***p <  = 0.0001; The color of the p-value symbol corresponds to the line it represents unless it is black in which case it pertains to the overall significance of all lines.

Overall, adenosine triphosphate (ATP)-Linked Respiration significantly increased as 2,3-dimethoxy-1,4-napthoquinone (DMNQ) concentration increased [F(2,18) = 33.87, p < 0.0001; 5 uM DMNQ t(27) = 8.23, p < 0.0001; 10 uM DMNQ t(27) = 4.23, p = 0.0005] (Fig. 1A).

The effect of TCAH exposure on ATP-Linked Respiration was dependent on DMNQ concentration [F(6,224) = 8.14, p < 0.0001]. Without DMNQ exposure, ATP-Linked Respiration decreased, as compare to baseline, as TCAH concentration increased but this effect was not statistically significant (Fig. 1E). When exposed to 5 uM or 10 uM DMNQ, ATP-Linked Respiration increased as compared to baseline as TCAH was increased with 0.5 mM TCAH concentration showing statistical significance [5 uM DMNQ: t(224) = 2.24, p < 0.01; 10 uM DMNQ: t(224) = 2.10, p < 0.05] (Fig. 1E).

#### Proton Leak Respiration

Overall Proton Leak Respiration significantly increased as DMNQ concentration increased [F(2,18) = 49.67, p < 0.0001; 5 uM DMNQ t(27) = 8.98, p < 0.0001; 10 uM DMNQ t(27) = 8.20, p < 0.0001] (Fig. 1B).

The effect of TCAH exposure on Proton Leak Respiration was dependent on DMNQ concentration [F(6,224) = 9.67, p < 0.0001]. Without DMNQ exposure, Proton Leak Respiration did not change significantly as TCAH increased (Fig. 1F). When exposed to 5 uM or 10 uM DMNQ Proton Leak Respiration increased as TCAH concentration increased as compared to baseline; with 5 uM DMNQ this increase was significant at 0.5 mM TCAH [t(27) = 3.46, p < 0.001] and 1.0 mM TCAH [t(27) = 4.61, p < 0.0001] while with 10 uM DMNQ this increase was only significant at 0.5 mM TCAH [t(27) = 1.95, p < 0.05].

#### Maximal Respiratory Capacity

Overall Maximal Respiratory Capacity significantly decreased as DMNQ concentration increased [F(2,18) = 15.19, p < 0.0001; 10 uM t(27) = 5.18, p < 0.0001] (Fig. 1C).

The effect of TCAH exposure on Maximal Respiratory Capacity was dependent on DMNQ concentration [F(6,224) = 23.57, p < 0.0001]. Without DMNQ exposure, Maximal Respiratory Capacity significantly decreased, as compared to baseline, at the highest TCAH concentration [1.0 mM TCAH t(224) = 3.18, p < 0.005] (Fig. 1G). When exposed to 5 uM DMNQ Maximal Respiratory Capacity increased as TCAH concentration increased [0.5 mM TCAH t(27) = 2.91, p < 0.005; 1.0 mM TCAH t(27) = 2.71, p < 0.01]. A similar increase in Maximal Respiratory Capacity was also seen with 10 uM DMNQ although the increase was not significant.

#### Reserve Capacity

Overall Reserve Capacity significantly changed across DMNQ [F(2,18) = 70.30, p < 0.0001]. DMNQ exposure alone significantly reduced Reserve Capacity [5 uM DMNQ: t(27) = 8.00, p < 0.0001; 10 uM DMNQ: t(27) = 11.57, p < 0.0001] (Fig. 1D).

The effect of TCAH exposure on Reserve Capacity was dependent on DMNQ concentration [F(6,224) = 14.05, p < 0.0001]. Without DMNQ exposure Reserve Capacity significantly decreased, as compared to baseline, at the highest TCAH concentration [1.0 mM TCAH: t(224) = 5.80, p < 0.001] but when exposed to DMNQ Reserve Capacity was not significantly different than baseline for any TCAH concentration (Fig. 1H).

### Effect of TCAH on Mitochondrial Function in AD LCLs

The effects of TCAH on AD LCLs are reported in this section. Control findings from the previous section will not be repeated here, although the control LCL group is used as a comparison.

#### ATP-linked Respiration

**Figure 2:**
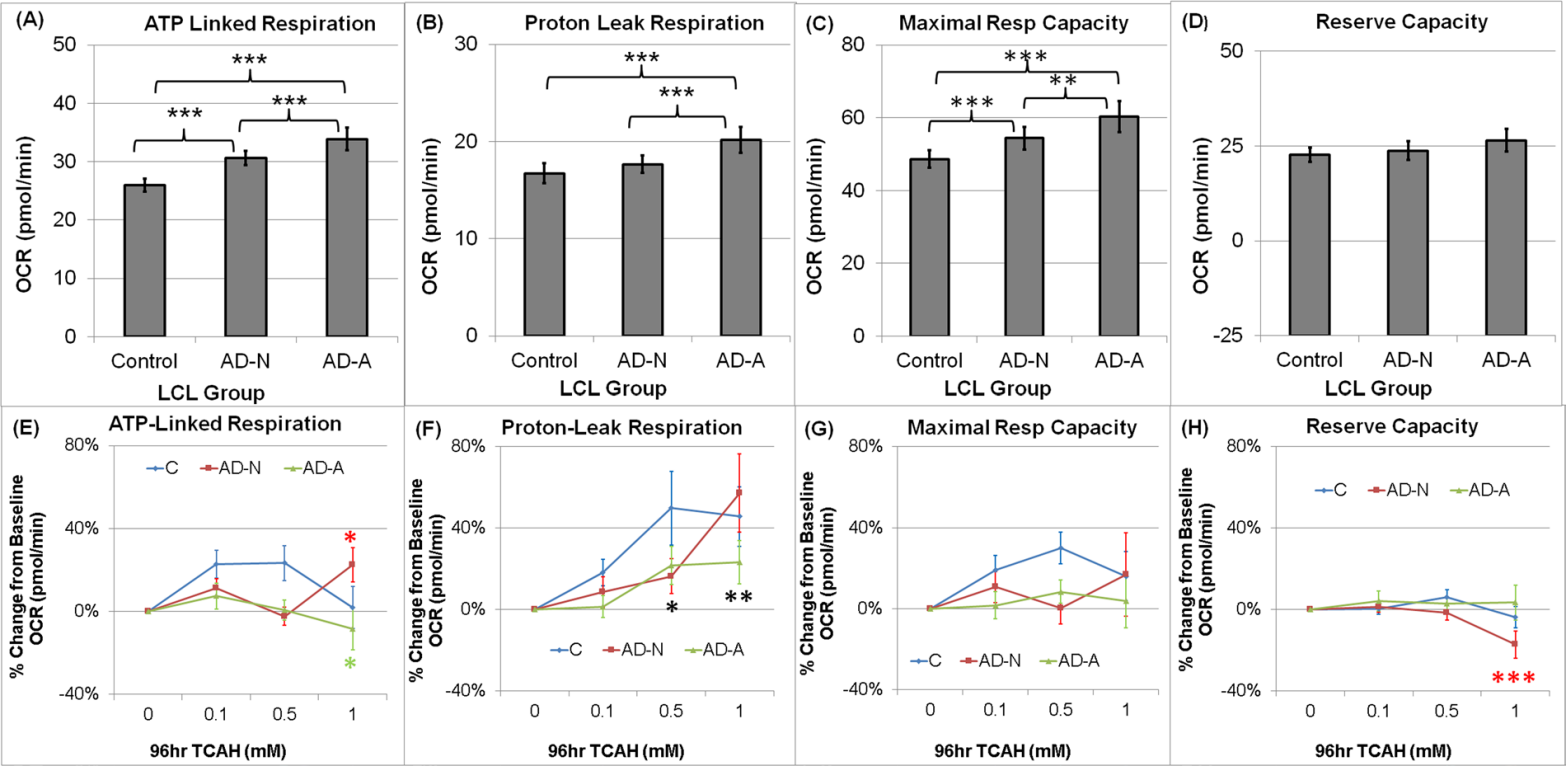
(**A**–**D**) Overall mitochondrial respiratory parameters for each LCL group and (**E**–**H**) Relative (percent) change from baseline in mitochondrial respiratory parameters for each lymphoblastoid cell line (LCL) across trichloroacetaldehyde hydrate (TCAH) concentrations. Overall (**A**) ATP-linked Respiration, (**B**) Proton Leak Respiration, (**C**) Maximal Respiratory Capacity and (**D**) Reserve Capacity for each LCL group. (**E**) Overall ATP-Linked Respiration slightly but significantly decreased by TCAH for AD-A LCLs and significantly increased by TCAH for AD-N LCLs; (**F**) Proton Leak Respiration increased significantly as TCAH increases for all LCL groups; (**G**) There was no significant change in Maximal Respiratory Capacity for any LCL group; (**H**) Reserve Capacity was stable for the Control and AD-A LCLs but decreases significantly at the highest TCAH concentration for the AD-N LCLs. The line color correspond to the group (Blue = Control LCLs; Red = AD-N LCLs; Green = AD-A LCLs). The color of the p-value symbol corresponds to the line it represents unless it is black in which case it pertains to the overall significance of all lines. ^†^p <  = 0.05; *p <  = 0.01; **p <  = 0.001; ***p <  = 0.0001.

ATP-Linked Respiration was significantly different across Groups [F(2,794) = 48.78, p < 0.0001] with AD-A LCLs having a higher ATP-Linked Respiration than AD-N [t(794) = 3.94, p < 0.0001] and Control [t(794) = 9.80, p < 0.0001] LCLs and AD-N having a higher ATP-Linked Respiration as compared to Control LCLs [t(794) = 6.01, p < 0.0001] (See Fig. 2A).

The effect of TCAH concentration on ATP-Linked Respiration was different across LCL groups [F(6,794) = 11.19, p < 0.0001]. At 1.0 mM TCAH ATP-Linked Respiration was significantly higher than baseline for the AD-N LCLs [t(794) = 2.23, p < 0.05] but significantly lower than baseline for AD-A LCLs [t(794) = 2.38, p < 0.02] (See Fig. 2E).

**Figure 3:**
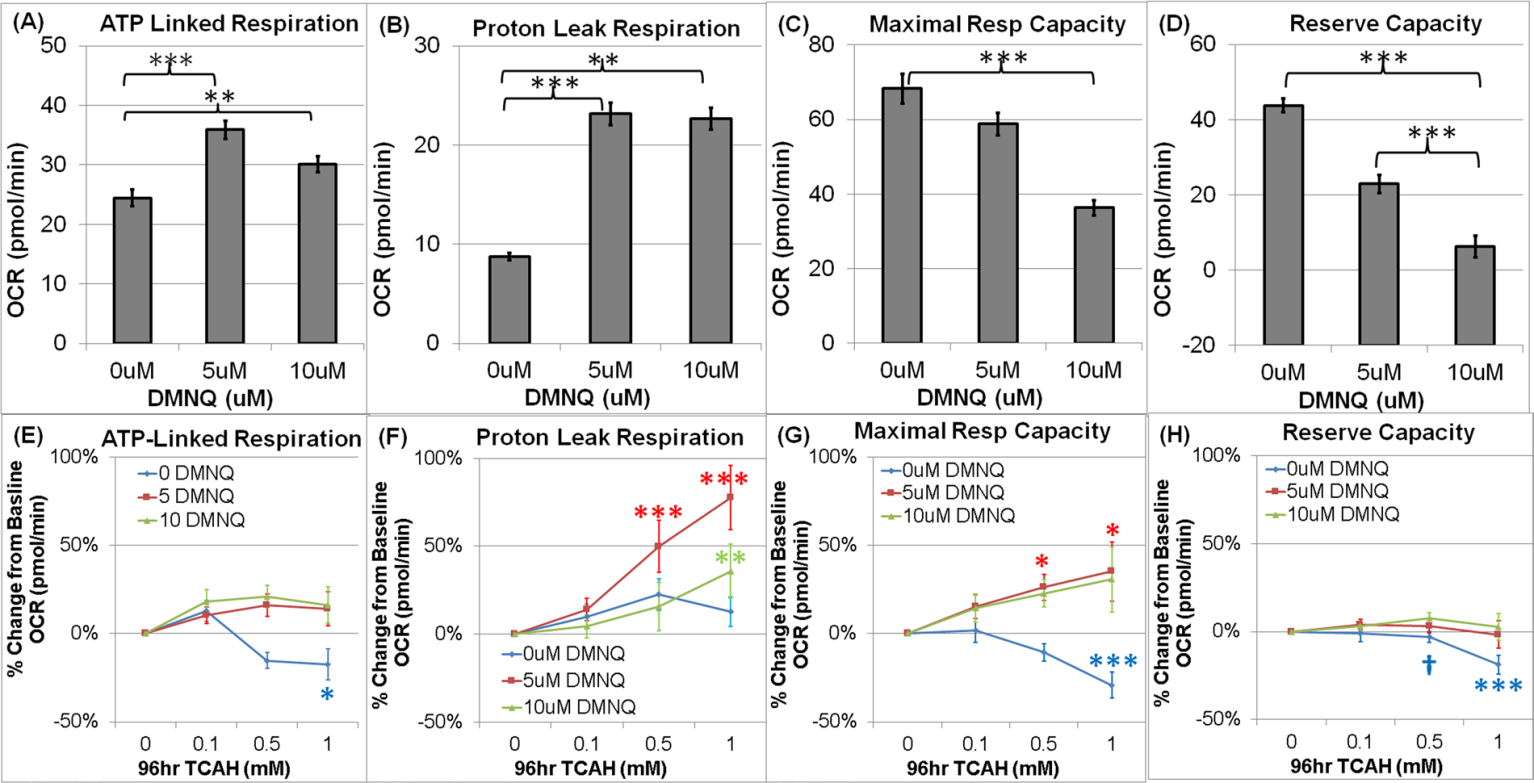
(**A**–**D**) Overall mitochondrial respiratory parameters for each DMNQ concentration and (**E**–**H**) Relative (percent) change from baseline in mitochondrial respiratory parameters for each 2,3-dimethoxy-1,4-napthoquinone (DMNQ) concentration across Trichloroacetaldehyde hydrate (TCAH) concentrations for *all lymphoblastoid cell lines* (*LCLs*) *combined*. Overall (**A**) ATP-linked Respiration, (**B**) Proton Leak Respiration, (**C**) Maximal Respiratory Capacity and (**D**) Reserve Capacity for each DMNQ concentration. (**E**–**H**) In general, mitochondrial respiratory parameters increased as TCAH concentration increased when TCAH exposure was followed by acute DMNQ exposure whereas mitochondrial respiratory parameters decreased as TCAH concentration increased when LCLs were not exposed to DMNQ. (**E**) ATP-Linked Respiration was stable across TCAH concentrations when LCLs were exposed to DMNQ after TCAH exposure but significantly decreased with increased TCAH concentrations when LCLs were not exposed to DMNQ; (**F**) Proton Leak Respiration significantly increased across TCAH concentrations when LCLs were exposed to DMNQ after TCAH exposure but did not significantly change with increasing TCAH concentrations when LCLs were not exposed to DMNQ; (**G**) Maximal Respiratory Capacity significantly increased across TCAH concentrations when LCLs were exposed to DMNQ after TCAH exposure but, in contrast, significantly decreased with increasing TCAH concentrations when LCLs were not exposed to DMNQ; (**G**) Reserve Capacity significantly decreased with increasing TCAH when LCLs were not exposed to DMNQ, but remained stable if TCAH was followed by DMNQ. The line color correspond to the DMNQ exposure (Blue = No DMNQ; Red = 5 uM DMNQ; Green = 10 uM DMNQ). The color of the p-value symbol corresponds to the line it represents unless it is black in which case it pertains to the overall significance of all lines. ^†^p <  = 0.05; *p <  = 0.01; **p <  = 0.001; ***p <  = 0.0001.

ATP-Linked Respiration was significantly different across DMNQ concentrations [F(2,18) = 36.94, p < 0.0001]. ATP-Linked Respiration was significantly higher at 5 uM DMNQ [t(18) = 8.59, p < 0.0001] and 10 uM DMNQ [t(18) = 4.39, p < 0.0005] as compared to baseline (Fig. 3A).

The effect of TCAH on ATP-Linked Respiration depended on DMNQ concentration [F(6,794) = 5.25, p < 0.0001]. In LCLs exposed to DMNQ ATP-Linked Respiration did not significantly change from baseline as TCAH increased (Fig. 3E). However, without DMNQ exposure, ATP-Linked Respiration decreased below baseline as TCAH increased with a significant depression at 1 mM TCAH [t(794) = 2.45, p = 0.01] (Fig. 3E).

#### Proton Leak Respiration

Proton Leak Respiration significantly differed across LCL Groups [F(2,794) = 17.81, p < 0.0001] with AD-A LCLs having higher Proton Leak Respiration as compared to AD-N [t(794) = 4.34, p < 0.0001] and Control [t(794) = 5.73, p < 0.0001] LCLs (Fig. 2B).

Overall Proton Leak Respiration significantly changed with TCAH concentration [F(3,27) = 6.85, p = 0.001] with Proton Leak Respiration being significantly higher at 0.5 mM TCAH [t(27) = 2.24, p < 0.05] and 1.0 mM TCAH [t(27) = 4.11, p < 0.0005] as compared to baseline (Fig. 2F).

Overall Proton Leak Respiration significantly changed with DMNQ exposure [F(2,18) = 60.43, p < 0.0001] with Proton Leak Respiration being significantly higher at 5 uM DMNQ [t(18) = 11.76, p < 0.0001] and 10 uM DMNQ [t(18) = 11.55, p < 0.0005] as compared to baseline (Fig. 3B). However, the effect of DMNQ on Proton Leak Respiration was different across LCL Groups [F(6,794) = 2.83, p < 0.05], primarily due to Proton Leak Respiration increasing to a lesser extent in AD-N and Control LCLs as compared to AD-A LCLs [Control vs AD-A: 5 uM t(794) = 3.24, p = 0.001; 10 uM: t(794) = 5.33, p < 0.0001; AD-N vs AD-A: 5 uM t(794) = 3.21,p = 0.001; 10 uM t(794) = 4.10, p < 0.001] (Data not shown).

The effect of TCAH on Proton Leak Respiration depended on DMNQ exposure [F(6,794) = 8.32, p < 0.0001] (Fig. 3F). There was no significant change in Proton Leak Respiration without DMNQ exposure whereas DMNQ exposure resulted in a significantly increased Proton Leak Respiration as TCAH increased with Proton Leak Respiration significantly greater than baseline at 0.5 mM TCAH [t(794) = 3.86, p < 0.0001] and 1.0 mM TCAH [t(794) = 6.37, p < 0.0001] TCAH for 5 uM DMNQ and at 1.0 mM TCAH [t(794) = 3.28, p = 0.001] with 10 uM DMNQ.

#### Maximal Respiratory Capacity

Maximal Respiratory Capacity was significantly different across LCL Groups [F(2,794) = 22.47, p < 0.0001]. Overall Maximal Respiratory Capacity was significantly higher for AD-A LCLs as compared to AD-N [t(794) = 3.22,p = 0.001] and Control [t(794) = 6.70,p < 0.0001] LCLs and for AD-N as compared to Control LCLs [t(794) = 3.58, p < 0.0001] (Fig. 2C).

Maximal Respiratory Capacity was significantly different across DMNQ concentrations [F(2,18) = 17.74, p < 0.0001] with Maximal Respiratory Capacity significantly declining as DMNQ concentration increased with Maximal Respiratory Capacity being significantly lower at the highest DMNQ concentration [t(18) = 5.81, p < 0.0001] as compared to no DMNQ exposure (Fig. 3C).

The effect of TCAH on Maximal Respiratory Capacity depended on DMNQ exposure [F(6,794) = 15.08, p < 0.0001] (Fig. 3G). Without acute exposure to DMNQ, increasing concentrations of TCAH resulted in a depression of Maximal Respiratory Capacity with this depression being significant at 1 mM TCAH [t(794) = 5.08, p < 0.0001]. However, with exposure to 5 uM and 10 uM DMNQ, Maximal Respiratory Capacity increased as TCAH increased with this effect being significant for 5 uM DMNQ [0.5 mM TCAH: t(794) = 2.59, p = 0.01; 1.0 mM TCAH: t(794) = 2.93, p < 0.005]. The same trend was seen for 10 uM DMNQ, but the increases were not statistically significant.

#### Reserve Capacity

Overall, Reserve Capacity was significantly affected by TCAH exposure [F(3,27) = 3.57,p < 0.05] with Reserve Capacity being significantly depressed at the highest TCAH concentration [t(27) = 2.29, p = 0.03]. However, this effect was different across LCL Groups [F(6,794) = 5.31, p < 0.0001] and was driven by the AD-N group (Fig. 2H) as demonstrated by three effects. First, Reserve Capacity did not change significantly from baseline for Control or AD-A LCLs as TCAH increased. Second, AD-N LCLs demonstrated a significant depression in Reserve Capacity at 1.0 mM TCAH [t(794) = 4.49,p < .0001]. Third, Reserve Capacity for AD-N LCLs was significantly lower than AD-A LCLs at 1.0 mM [t(794) = 4.79, p < 0.0001] and 0.5 mM [t(794) = 2.34, p < 0.05] TCAH and significantly lower than Control LCLs [t(794) = 2.41,p < 0.05] at 1.0 mM TCAH. In addition, Reserve Capacity for AD-A LCLs was higher than Control LCLs at 1.0 mM [t(794) = 2.34,p < 0.05].

Reserve Capacity significantly decreased with DMNQ exposure [F(2,18) = 40.52, p < 0.0001; 5 uM DMNQ: t(18) = 6.05, p < 0.0001; 10 uM DMNQ: t(18) = 8.80, p < 0.0001] (Fig. 3D). However, the effect of DMNQ on Reserve Capacity was different across LCL Groups [F(4,794) = 9.20, p < 0.0001]. The DMNQ by Group interaction was driven by the fact that AD-A LCLs demonstrated a higher baseline Reserve Capacity and a more significant decline in Reserve Capacity with increased DMNQ, recapitulating the findings from our previous studies (Supplementary Figure 1D).

The effect of TCAH on Reserve Capacity was also dependent on DMNQ concentration [F(6,794) = 11.25, p < 0.0001]. As seen in Fig. 3H, TCAH alone significantly decreased Reserve Capacity at 0.5 mM TCAH [t(794) = 2.27, p = 0.02] and 1.0 mM TCAH [t(794) = 5.99, p < 0.0001], however, Reserve Capacity did not change across TCAH concentrations when cells were exposure to DMNQ.

**Figure 4:**
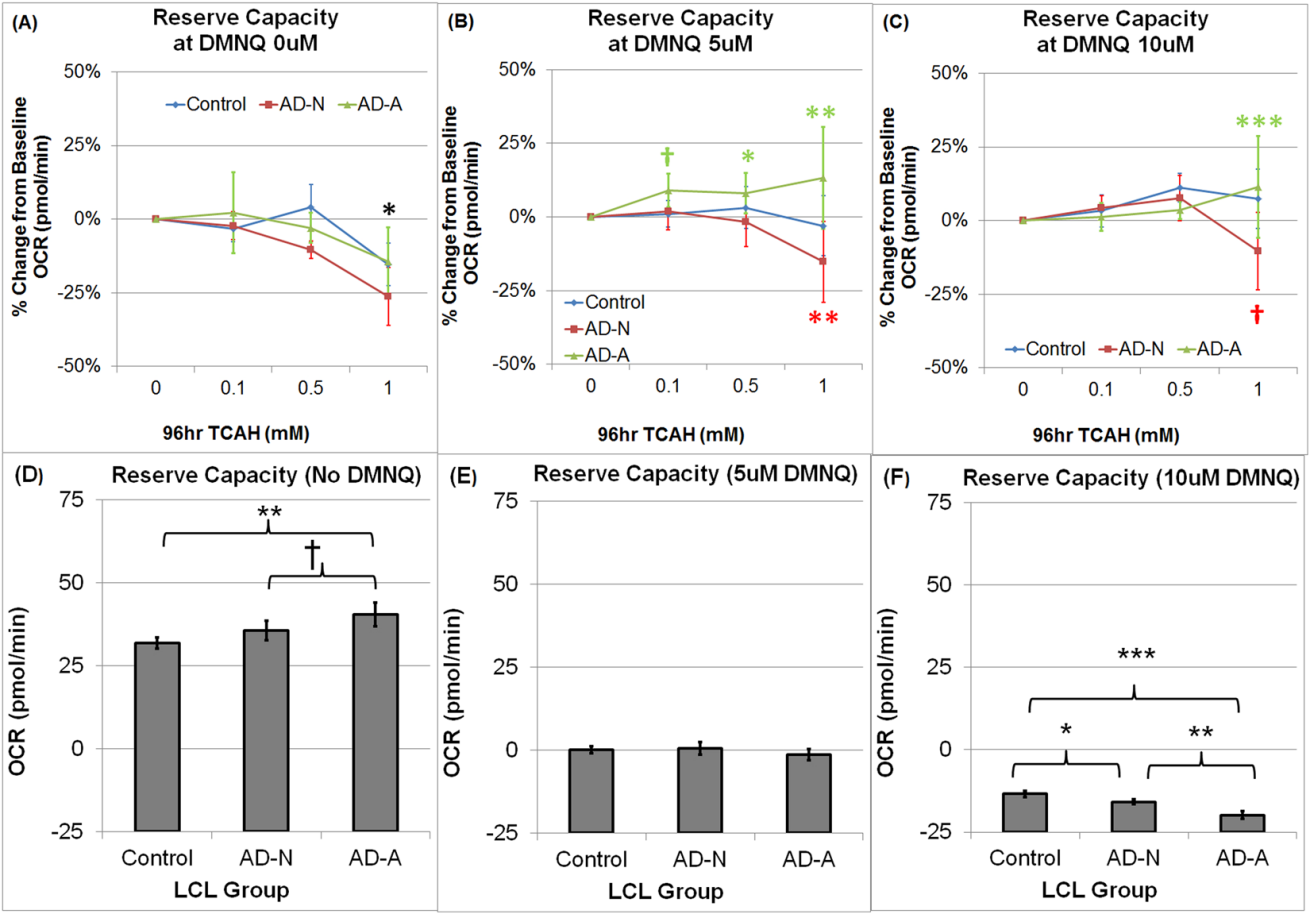
Relative (percent) change from baseline in Reserve Capacity as trichloroacetaldehyde hydrate (TCAH) concentration increased for each 2,3-dimethoxy-1,4-napthoquinone (DMNQ) concentration by lymphoblastoid cell line (LCL) group and overall Reserve Capacity by LCL group. In general, with DMNQ exposure, AD-N LCLs demonstrate a loss of Reserve Capacity as TCAH concentration increases while AD-A LCLs demonstrate an increase in Reserve Capacity as TCAH concentration increases (**A**) Without DMNQ Exposure, Reserve Capacity decreases as TCAH increases with the highest dose of TCAH demonstrating a significant depression in Reserve Capacity. (**B**) With 5 uM DMNQ Reserve Capacity increases for AD-A LCLs with TCAH exposure but decreases for AD-N LCLs with TCAH exposure. (**C**) With 10 uM DMNQ Reserve Capacity increases for AD-A LCLs at the highest TCAH Concentration but decreases for AD-N LCLs at the highest TCAH Concentration. The line color correspond to the group (Blue = Control LCLs; Red = AD-N LCLs; Green = AD-A LCLs). The color of the p-value symbol corresponds to the line it represents unless it is black in which case it pertains to the overall significance of all lines. (**D**) Without DMNQ, AD-A LCLs demonstrate the highest Reserve Capacity, as we have demonstrated in previous studies. (**E**) With 5 uM DMNQ exposure, there is no difference in Reserve Capacity across LCL groups. (**F**) With 10 uM DMNQ Control LCLs demonstrate a slightly but significantly higher Reserve Capacity than other LCL groups. ^†^p <  = 0.05; *p <  = 0.01; **p <  = 0.001; ***p <  = 0.0001.

The modifying effect of DMNQ on the influence of TCAH on Reserve Capacity was different for each LCL Group [F(12,794) = 1.76, p = 0.05]. To examine this TCAH by DMNQ by Group interaction, an analysis was conducted on Reserve Capacity for each DMNQ concentration. With no DMNQ exposure (Fig. 4A), Reserve Capacity significantly decreased as TCAH concentration increased [F(3,27) = 5.49, p < 0.005] such that Reserve Capacity was significantly depressed at the highest TCAH concentration [t(27) = 3.20, p < 0.005]. In addition, Reserve Capacity was significantly different across Group [F(2,269) = 7.14, p < 0.001] with AD-A LCLs having a significantly higher Reserve Capacity as compared to AD-N [t(269) = 2.24, p < 0.05] and Control [t(269) = 3.76, p < 0.0005] LCLs (Fig. 4D).

With 5 uM DMNQ exposure (Fig. 4B), the effect of TCAH on Reserve Capacity was significantly different across Group [F(6,189) = 6.85, p < 0.0001]. For the Control LCLs, Reserve Capacity did not change as TCAH increased while AD-A LCLs demonstrated an increase in Reserve Capacity as TCAH increased [0.1 mM TCAH: t(189) = 2.16, p < 0.05; 0.5 mM TCAH: t(189) = 2.89, p < 0.005; 1.0 mM TCAH: t(189) = 3,74, p < 0.0005]. In contrast, Reserve Capacity significantly decreased as TCAH increased for AD-N LCLs with this decrease being significant at 1.0 mM TCAH [t(189) = 3.29, p = 0.001].

With 10 uM DMNQ exposure (Fig. 4C), the effect of TCAH on Reserve Capacity was different for each LCL Group [F(6,282) = 8.84, p < 0.0001]. For the Control LCLs, Reserve Capacity did not change as TCAH increased while Reserve Capacity increased as TCAH increased for AD-A LCLs such that Reserve Capacity was significantly elevated at 1.0 mM TCAH [t(282) = 4.05, p < 0.0001]. In contrast, Reserve Capacity significantly decreased as TCAH increased for AD-N LCLs such that Reserve Capacity was significantly lower at 1.0 mM TCAH [t(282) = 3.70,p = 0.02]. With 10 uM DMNQ, Reserve Capacity was also different across LCL Group [F(2,282) = 19.18, p < 0.0001]. Control LCLs had a significantly higher Reserve Capacity as compared to the AD-N [t(282) = 2.67, p < 0.01] and AD-A [t(282) = 6.17, p < 0.0001] LCLs and AD-N demonstrated a higher Reserve Capacity as compared to AD-A LCLs [t(282) = 3.64, p = 0.001] (Fig. 4F).

## Discussion

This study examined mitochondrial respiratory function in lymphoblastoid cells (LCLs) derived from children with autism spectrum disorder (ASD) and age and gender-matched controls after exposure to trichloroacetaldehyde hydrate (TCAH), a metabolite of a known toxicant, trichloroethylene (TCE). TCE is known to cause neurotoxic^15^, mitochondrial^16–19^, redox^20, 21^ and immune^22–25^ abnormalities in animals and has been linked to neurological disorders in humans, specifically ASD^6, 32^ and Parkinson’s Disease^16, 19, 30, 31^. TCAH has been shown to have physiological effects similar to TCE^22–25^.

The LCLs derived from children with ASD used in this study were previously characterized into two types: AD-N LCLs which have mitochondrial function similar to Control LCLs, and AD-A LCLs which have atypical mitochondrial function characterized by an elevated Reserve Capacity at baseline and vulnerability to acute increases in ROS^41, 42^. We hypothesized that AD-A LCLs adapted their respiratory function to be more resilient to environmental toxicants, presumably due to previous exposure to environmental stressors. This study supports this hypothesis.

### The Effect of TCAH on Mitochondrial Function

Overall, for all LCLs without DMNQ exposure, ATP-Linked Respiration and Maximal Respiratory Capacity, both indices of ETC integrity and function, were reduced as TCAH concentration increased whereas Proton Leak Respiration did not increase, suggesting that TCAH did not increase mitochondrial ROS production (Fig. 3F). Reserve Capacity remained stable until the two highest TCAH concentrations where it significantly decreased. Altogether, these data suggest prolonged exposure to TCAH does negatively influence mitochondrial function in LCLs at the higher end of the concentrations used in this study, with this effect primarily due to inhibiting or damaging the ETC rather than increasing mitochondrial ROS. This effect is consistent with previous studies which have associated TCE with mitochondrial metabolism abnormalities^16–18, 48^.

Although mitochondrial ROS, as indexed by Proton Leak Respiration, did not have a role in mitochondrial dysfunction during the Seahorse assay, given that ROS have been associated with TCE and TCAH^20^, it is possible that ROS could have had a role in damaging the ETC during the 96 hr TCAH exposure with this effect persisting after TCAH was washed from the LCLs.

Both AD-A and Control LCLs showed a stable Reserve Capacity as TCAH concentration increased (Fig. 2H). In contrast, AD-N LCLs showed a significant decreased in Reserve Capacity as TCAH increased. This was due to the fact that ATP-Linked Respiration and Proton Leak Respiration increased without a compensatory increase in Maximum Reserve Capacity in AD-N LCLs. Thus, it appears that the AD-A LCLs, overall, acted like Control LCLs for most mitochondrial parameters. Both AD-A and Control LCLs appear to have compensatory mechanisms to prevent a decrease in Reserve Capacity at the highest concentration of TCAH. This suggests that the AD-N LCLs are the most sensitive to TCAH under normal conditions. This supports our hypothesis that the AD-A LCLs have adapted their mitochondrial function to maintain a more normal physiology in the face of intrinsic and extrinsic stressors, potentially through mitoplasticity.

### The Effect of Acute DMNQ following TCAH exposure on Mitochondrial Function

When acute DMNQ exposure followed TCAH exposure in Control LCLs, ATP-Linked and Maximum Respiratory Capacity increased, which is in contrast to the effect of TCAH without DMNQ (Fig. 1E and G). DMNQ exposure itself increased ATP-Linked Respiration with this effect being potentiated by TCAH incubation. DMNQ itself reduced Maximal Respiratory Capacity but TCAH exposure reversed this decrease. Thus, there appears to be an increase in ETC function induced by prolonged TCAH exposure when followed by an acute DMNQ exposure.

For Control LCLs, DMNQ itself significantly increased Proton Leak Respiration with TCAH treatment potentiating this increased but only for the 5 uM DMNQ exposure (Fig. 1B and F). Unlike the decrease in Reserve Capacity with increasing TCAH concentrations found without DMNQ exposure, Reserve Capacity showed no significant change with increasing TCAH concentration when exposed to DMNQ. This suggests the increase in ATP-Linked Respiration and Maximal Respiratory Capacity induced by the acute DMNQ exposure may have induced a protective mechanism to the cell, over and above the increase in Proton Leak Respiration, in order to prevent a decrease in Reserve Capacity.

The effect of DMNQ on Control LCLs was similar when examining LCLs overall (Fig. 3). That is, acute DMNQ exposure following TCAH exposure reversed the reduction in ATP-Linked Respiration (Fig. 3E) and Maximum Respiratory Capacity (Fig. 3G) seen with increasing TCAH concentration without DMNQ exposure, resulting in a stable ATP-Linked Respiration and an increase in Maximal Reserve Capacity with increasing TCAH concentrations. Similarly, overall, DMNQ significantly increased Proton Leak Respiration with TCAH potentiating this effect (Fig. 3F) and TCAH did not induce any change in Reserve Capacity if followed by DMNQ (Fig. 3H). These findings point to the notion that prolonged TCAH exposure induces adaptive changes to the systems which protect the cell against ROS. However, it is noted that this potentiating effect of TCAH seems to be most robust at 5 uM DMNQ, suggesting a plateau in the ability of TCAH to induce protective adaptive changes in the cell; perhaps the increased ROS associated with 10 uM DMNQ starts to overwhelm the protective mechanisms induced.

### Effect of DMNQ Following TCAH Exposure in AD LCLs

The potentiating effect of DMNQ on TCAH exposure for Reserve Capacity was different across LCL groups. When exposed to DMNQ, Control LCLs did not demonstrate a decline in Reserve Capacity as TCAH increased, while AD-N LCLs demonstrated a significant reduction in Reserve Capacity as TCAH increased (Fig. 4A–C). In contrast, AD-A LCLs demonstrated a significant increase in Reserve Capacity as TCAH increased. Although the AD-A LCLs demonstrated a lower absolute Reserve Capacity, these findings demonstrate that exposure to TCAH enhances the protective mechanisms to ROS in the AD-A LCLs. This supports our hypothesis that AD-A LCLs are adapted to function in the context of environmental toxicants such as TCAH.

Control LCLs after TCAH incubation followed by DMNQ exposure demonstrated a relative increase in ATP-Linked Respiration, Proton Leak Respiration and Maximal Reserve Capacity. Similarly, AD-A LCLs show above normal values for these respiratory parameters at baseline. This suggests that the changes in baseline respiratory function of the AD-A LCLs could be adaptive changes that resulted from environmental exposures^42^. ASD LCLs have intrinsic increases in ROS^42, 49^, raising the possibility of an intrinsic chronic ROS stimulus which may be stimulating adaptive changes in respiratory function. The source of the chronic ROS increases in ASD LCLs is not known, but studies examining ETC enzymes in ASD immune cells show that Complex I and III produced approximately twice as much hydrogen peroxide and 1.6 times more mitochondrial ROS as controls^43, 44^.

In addition, N-acetyl cysteine (NAC), a precursor to glutathione which can decrease ROS, protects the AD-A LCLs from a decline in Reserve Capacity with increased DMNQ^41^ and protects mice from TCE-induced autoimmunity^50^, suggesting that increased ROS might be a driving force for both the intrinsic abnormalities in AD-A LCLs and the toxic effects of TCE.

### Possible TCAH Induced Mitoplasticity

The mechanisms accounting for changes in mitochondrial respiration when DMNQ follows TCAH exposure are consistent with mitoplasticity. Mitoplasticity refers to the mitochondria’s ability to adapt or transform to optimally function in the face of changes in energy demand, substrate availability, pathophysiology or environmental stressors^5^. Mitoplasticity involves pathways that modulate general cellular energetics, mitochondrial enzymes in the citric acid cycle and ETC, proton leak, redox regulation and transcription factors as well as genes that regulate the control of mitochondrial repair and regeneration such as mitochondrial biogenesis, mitophagy and mitochondrial fission and fusion^5^.

Proton Leak Respiration represents a mechanism to compensate for increased ROS at the inner mitochondrial membrane and is mediated by Uncoupling Protein 2 (UCP2) in lymphocytes. UCP2 is known to be up-regulated in the context of prolonged oxidative stress and serves to protect the mitochondria^51, 52^, AD-A LCLs have increased UCP2 protein as compared to AD-N LCLs^42^, which is consistent with our findings that the increase in Proton Leak Respiration was greater in the AD-A as compared to the AD-N LCLs. One study showed that sub-acute TCE exposure up-regulates UCP2 gene expression^29^ and other studies have demonstrated that sub-acute exposure to TCE up-regulates PGC1α^27, 28^ which has a downstream effect of up-regulating UCP2. PGC1α also has the effect of upregulating ETC complex genes as well as mitochondrial DNA transcription. Such effects may account for the increases in ATP-Linked Respiration and Maximal Respiratory Capacity seen with the combination of TCAH and DMNQ exposure.

The probable up-regulation of mitoplasticity pathways by TCAH exposure suggests that TCAH influences pathways which respond to metabolic stresses. Several studies have documented that sub-acute TCE exposure induces epigenetic changes in specific genes including those involved in DNA methylation^29^. The 96 hr TCAH exposure time used in this study is within the time frame in which epigenetic changes can occur. Future studies may focus on epigenetic changes in genes involved in mitoplasticity with TCE or TCAH exposure.

These findings suggest that expression of mitoplasticity may require an acute stressor in order to activate underlying potentiated pathways. Studies that have measured ETC complex function found that TCE exposure was detrimental on mitochondrial function but these studies measured ETC function in isolated mitochondria. By using isolated mitochondria, cellular regulatory pathways that could enhance mitochondrial function were not active during such measurements of mitochondrial function. Our data suggests that a metabolic stressor, such as increased ROS, may be needed to activate mitoplasticity pathways. This supports the notion that protective mitochondrial mechanisms may need to be uncovered by physiological stressors in order to measure their full functional consequences.

### Application to Mitochondrial Dysfunction in ASD

Mitochondrial dysfunction is a major physiological disturbance associated with ASD^9^. The mitochondria could be a key mediator by which environmental stressors increase the risk of developing ASD as mitochondrial function can be compromised by environmental exposures implicated in ASD such as heavy metals^53–56^, exhaust fumes^57^, polychlorinated biphenyls^58^ and pesticides^59, 60^. Alternatively, endogenous stressors associated with ASD such as elevated pro-inflammatory cytokines^61–63^ and increased oxidative stress^64, 65^ can also damage and decrease mitochondrial function.

Some of the data supporting the notion of mitochondrial dysfunction in ASD appears contradictory. Most direct measurements of ETC enzyme function has traditionally found decreases in ETC function^9, 43, 44^ yet other studies suggest that ETC function may be increased rather than decreased in some cases^39, 41, 42, 45, 66–69^. This study suggests that ETC function may be dependent on whether mitoplasticity pathways are engaged. This also suggests that testing enzyme function in isolated mitochondria without supportive cellular pathways may not provide a true reflection of how the mitochondrial may function *in vivo*.

The heterogeneity of the ASD population could make the association of specific mitoplasticity genes with ASD difficult to detect considering that probably only a subset of individuals with ASD have mitochondrial dysfunction^9, 41, 42^. However, some mitoplasticity genes have been associated with ASD. For example, we have shown that UCP2 is increased in the AD-A LCLs as compared to the AD-N LCLs in our previous study, accounting for higher proton leak respiration in the AD-A LCLs^42^. Another study has linked ASD with uncoupling protein 4 (UCP4), an isoform predominately expressed in the central nervous system^70^. In neurons from Tuberous Sclerosis mice, a model of ASD, UCP2^71^ and AMPK^72^ were found to be up-regulated. Tuberous Sclerosis is associated with up-regulation in mTOR which enhances mitochondrial function and inhibits autophagy^5^. There are also several links between ASD and PGC1α. PGC1α is associated with modulation of the excitatory-inhibitory balance in the hippocampus such that elevated PGC1α could account for the cortical hyperexcitability associated with the ASD brain^73^. Thus, although more research is needed to support the link between the upregulation of mitoplasticity genes and ASD, there is evidence to support this notion.

### Limitations

A limitation inherent in ASD research is the availability of sufficient biological samples and the limitation in the models available to study such a heterogeneous disorder. Since there are no animal models that encompass the complete phenotype of ASD, especially the phenotype associated with mitochondrial dysfunction, we have developed a LCL based model.

Mitochondrial dysfunction preferentially affects high energy demanding systems, particularly the brain and immune system; thus, immune cells may be an ideal surrogate for investigating the consequences of mitochondrial abnormalities when neural tissue cannot be practically studied. However, animal or neural cell models with similar mitochondrial abnormalities could be helpful to examine the effect of mitochondrial dysfunction and mitoplasticity on behavior and neuronal function. In addition, we have discussed mitoplasticity, epigenetic mechanism and redox metabolism in this study. Future studies should examine changes in the expression of genes involved in mitochondrial fission and fusion, biogenesis and mitoplasticity, measure redox metabolism and screen for epigenetic changes to support these proposed mechanisms.

## Conclusions

This study demonstrates several effects of TCAH on mitochondrial function. First, TCAH appears to have a detrimental effect on the mitochondria, consistent with previous studies, although this effect is different across cell types with some LCLs derived from ASD children being more vulnerable than others. Second, when exposure to TCAH is followed by an oxidized microenvironment the detrimental effect of TCAH is reversed in some cell types, specifically Controls and AD-A LCLs. Third, we previously suggested that the AD-A LCL subgroup of ASD LCLs had adapted their mitochondrial function to be more resistant to environmental stressors. This study provides support for this notion. We demonstrated that AD-A LCLs were indeed more resistant to the effects of TCAH and we demonstrated that some of the adaptive changes that occurred in the Control LCLs due to exposure to TCAH were similar to the changes seen in AD-A LCLs at baseline. We interpret these findings in the context of mitoplasticity and have outlined how previous studies on the effect of TCAH have found that genes considered within mitoplasticity pathways are induced by TCAH. This provides insight into an aspect of atypical mitochondrial dysfunction seen in a subset of LCLs derived from children with ASD and may provide insight into metabolic disorders in a subset of individuals with ASD as well as the etiology of these abnormalities. In addition, further research may investigate the use of AD-A LCLs as a model of mitoplasticity, particularly mitoplasticity induced by environmental agents.

## Methods

### Lymphoblastoid Cell Lines and Culture Conditions

Seventeen LCLs derived from white males diagnosed with non-syndromic ASD chosen from pedigrees with at least 1 affected male sibling (mean/SD age 8.4 ± 4.0 y) were obtained from the Autism Genetic Resource Exchange (Los Angeles, CA, USA) and the National Institutes of Mental Health (Bethesda, MD, USA) center for collaborative genomic studies on mental disorders (Table 1). Seven of the ASD LCLs were identified as AD-A and 10 AD-N, as characterized in our previous studies^41, 42^. Because of the limited number of AD-A LCLs that were available to age match, some AD-A LCLs were matched twice. Ten age-matched control LCLs (mean/SD age 8.7 ± 4.2 y) were obtained from Coriell Cell Repository (Camden, NJ, USA). The control LCLs were obtained from healthy white males with no behavioral or neurological disorders or first-degree relatives with a medical disorder that could involve abnormal mitochondrial function. Cells were studied at an average passage 12, with a maximum passage of 15 since genomic stability is very high at this low passage number^74, 75^. Cells were maintained in RPMI 1640 culture medium with 15% FBS and 1% penicillin/streptomycin (Invitrogen, Grand Island, NY, USA) in a humidified incubator at 37 °C with 5% CO_2_.

**Table 1:** Lymphoblastoid cell line characteristics and matching between AD and control cell lines. List is organized by the two groups identified: AD-A and AD-N.

Pair #	AD-N LCLs			AD-A LCLs			Control LCLs		
	Cell ID	Source	Age (y)	Cell ID	Source	Age (y)	Cell ID	Source	Age (y)
1	01C08367	NIMH	7	01C08594	NIMH	7	GM09642	Coriell	7
2	1215301	AGRE	12	03C16499	NIMH	11	GM16007	Coriell	12
3	03C14349	NIMH	17	939303	AGRE	11	GM17272	Coriell	17
4	008404	AGRE	13	1165302	AGRE	13	GM11626	Coriell	13
5	01C08022	NIMH	5	01C08495	NIMH	4	GM09380	Coriell	6
6	02C09650	NIMH	7	02C09713	NIMH	7	GM11973	Coriell	7
7	1267302	AGRE	10	03C16499	NIMH	11	GM10153	Coriell	10
8	02C10054	NIMH	6	01C08594	NIMH	7	GM17255	Coriell	6
9	03C15992	NIMH	5	01C08495	NIMH	4	GM18054	Coriell	5
10	04C24363	NIMH	4	1393306	AGRE	3	GM09659	Coriell	4

NIMH = National Institutes of Mental Health (Bethesda, MD, USA).

AGRE = Autism Genetic Resource Exchange (Los Angeles, CA, USA).

Coriell = Coriell Cell Repository (Camden, NJ, USA).

### Seahorse Assay

**Figure 5:**
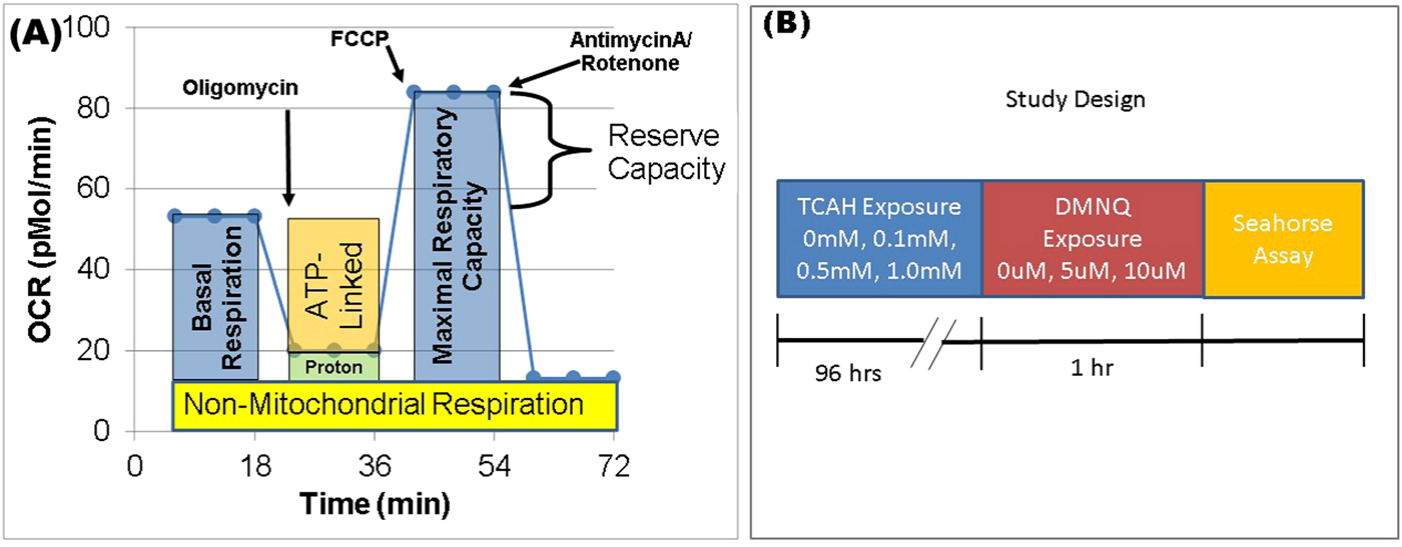
The Seahorse Assay and Experiment Timeline. (**A**) Parameters of mitochondrial respiration were derived by the sequential addition of pharmacological agents to respiring cells. For each parameter, three oxygen consumption rate (OCR) measurements are made over 18 minutes. First, baseline OCR is measured, from which Basal Respiration is derived by subtracting non-mitochondrial respiration. Next oligomycin, a complex V inhibitor, is added; the resulting OCR is used to derive ATP-Linked Respiration (by subtracting the oligomycin OCR from baseline OCR) and Proton Leak Respiration (by subtracting non-mitochondrial respiration from the oligomycin OCR). Next carbonyl cyanide-p-trifluoromethoxyphenyl-hydrazon (FCCP), a protonophore, is used to collapse the inner membrane gradient, driving ETC function to its maximal rate; Maximal Respiratory Capacity is derived by subtracting non-mitochondrial OCR from the FCCP OCR. Lastly, antimycin A, a complex III inhibitor, and rotenone, a complex I inhibitor, are added to shut down ETC function, revealing the non-mitochondrial respiration. Reserve Capacity is calculated by subtracting Basal Respiration from Maximal Respiratory Capacity (**B**) Timeline for the experiment. Lymphoblastoid cell lines (LCLs) are exposed to one of three concentrations of trichloroacetaldehyde hydrate (TCAH) or not exposed (baseline control) for 96 hrs. Following TCAH exposure, the LCLs are exposed to 2,3-dimethoxy-1,4-napthoquinone (DMNQ) for 1 hr in order to increase reactive oxygen (ROS) species or not exposed to DMNQ. The Seahorse assay is performed after these exposures.

We used the state-of-the-art Seahorse Extracellular Flux (XF) 96 Analyzer (Agilent Technologies, Santa Clara, CA USA), to measure the oxygen consumption rate (OCR), an indicator of mitochondrial respiration (Fig. 5A) in real-time in live intact LCLs.

One hour prior to the assay, cells were seeded onto poly-D-lysine coated 96-well XF-PS plates at a density of 1.1 × 10^5^ cells/well in DMEM XF assay media (unbuffered DMEM supplemented with 11 mM glucose, 2 mM L-glutamax, and 1 mM sodium pyruvate). Four replicate wells were used per treatment group. Titrations were performed to determine the optimal concentrations of pharmacological agents [oligomycin (1.0 µM), FCCP (0.3 µM), antimycin A (0.3 µM) and rotenone (1.0 µM)] used in the assay.

The Seahorse assay provides parameters of mitochondrial function and adenosine triphosphate (ATP) production. Both **ATP**-**Linked Respiration** and **Maximal Respiratory Capacity** provide information about the integrity and ability of the ETC to function. **Proton Leak Respiration** represents a control mechanism for minimizing ROS at the inner mitochondrial membrane, the site of the ETC complexes. Proton leak reduces the mitochondrial membrane potential which, in turn, decreases ETC ROS generation^76^. Proton Leak can be increased because of increased ROS at the inner mitochondrial membrane and/or because adaptive mechanisms have up-regulated this mechanism due to compensate for chronic increased ROS.

**Reserve Capacity** provides an index of mitochondrial and cellular health. **Reserve Capacity** becomes reduced as mitochondria reach their maximal functional capacity. At that point the mitochondria cannot provide additional ATP to the cell if needed, leaving the cell vulnerable. Both neurodegenerative^77, 78^ and heart^79^ diseases have been linked to depleted **Reserve Capacity** as has aging^80^. **Reserve Capacity** depletion is associated with cell death in cardiomyocytes^81^ and endothelial cells^82^ under conditions of oxidative stress and in neurons during glutamate toxicity or ETC inhibition^78, 83^.

### Redox Challenge

ROS was increased *in vitro* by exposing cells to increasing concentrations of the redox cycling agent, DMNQ (Sigma-Aldrich, St. Louis, MO, USA), for 1 h prior to the Seahorse assay (Fig. 5B). DMNQ generates both superoxide and hydrogen peroxide similar to levels generated by NADPH oxidase *in vivo* ^82^. A 5 mg/mL DMNQ solution was diluted in DMEM XF assay media into 10X stocks and added to cells in an XF-PS plate and incubated for 1 h at 37 °C in a non-CO_2_ incubator. The concentrations of 5 μM and 10 μM DMNQ were used to optimally increase ROS *in vitro* ^42^.

### Trichloroacetaldehyde hydrate exposure

Each group of LCLs were cultured with three concentrations of TCAH (0.1, 0.5 and 1.0 mM) for 96 hrs prior to the Seahorse assay (Fig. 1B) or cultured for the same length of time without TCAH exposure. The TCAH exposure length is equivalent to sub-acute exposure of TCE in animal models^27, 28^. These concentrations were selected to parallel our previous *in vitro* study that demonstrated that TCAH is an environmental autoimmune trigger^22^. Furthermore, acute exposure to TCE has been reported to result in 0.64 mM blood levels^84^, a concentration that is between the two highest concentrations used in this study. In addition, in mice a dose of 200 ppm TCAH resulted in a blood concentration of 0.2 mM^85^. Thus, the concentrations we used in our study are well within those documented in the blood of humans and animals.

### Analytic approach

Analysis of variance was conducted using a mixed-model regression^86^ via SAS version 9.3 (Cary, NC, USA) ‘glmmix’ procedure. The mixed-model compared matched AD LCL groups to each other and to the matched control LCLs, all of which were run in the same Seahorse assay plate. The mitochondrial respiratory measurement was the response variable with a between-group effect (e.g., AD-A v AD-N v control) and within-group repeated factor of DMNQ and TCAH concentration (modeled as a multilevel factor) as well as the interaction between these effects. We present the *overall* difference between the comparison groups (Group Effect), the overall effect of the DMNQ concentration (DMNQ effect), the overall effect of the TCAH concentration (TCAH effect) and the two- and three-way interactions between these factors. Random effects included the intercept, Seahorse Plate, TCAH and DMNQ, where applicable. When two-way interactions were significant, each concentration of TCAH was compared to the baseline value (i.e., values without exposure to TCAH) for each level of the other factor (e.g., each group). For the three-way interaction, a two-way analysis was performed for each DMNQ concentration. F-tests were used to evaluate significance of model statistics and are mentioned first in the results narrative to outline the overall effects. Planned post-hoc contrasts were t-distributed and are presented after the main effects to describe the specific comparison of a levels of an effect (i.e., TCAH 0.1 mM,0.5 mM, 1.0 mM or DMNQ 5 uM,10 M) to baseline.

To illustrate the effect of TCAH, the respiratory parameters are graphed as percent change from baseline. In order to obtain an accurate percent change calculation for Reserve Capacity, the Percent Reserve Capacity was used rather than the absolute Reserve Capacity. This was necessary because the absolute Reserve Capacity can be very small or even negative, which can result in invalid calculations when examining percent change. Percent Reserve Capacity was calculated by dividing Maximal Respiratory Capacity by Basal Respiration.

For the first set of analyses, we examine the effect of TCAH and DMNQ only on the Control LCLs in order to better understand the effect of TCAH on non-diseased cells. Second, we examine the effect TCAH and DMNQ on the two sets AD LCLs as well as Controls to understand the variation in the way the mitochondria responds to TCAH in the two different types of AD LCLs.

In addition, as part of supplemental material, we confirm the characteristics of the AD subgroups. Since we previously found that mitochondrial respiration in AD-A LCLs was different than AD-N and Control LCLs and that the latter two groups were similar to each other, planned post-hoc orthogonal contrasts are used to compare the AD-A to both the AD-N and Control LCLs combined and to compare AD-N and Control LCLs to each other (See Supplementary Material).

### Data Availability

Data is available upon request.

### Ethical Approval

This study uses deidentified cell lines and has been determined to be exempt from IRB review.

### Ethical Review

This research uses deidentified tissue that has been determined to be exempt from review by the local university Institutional Review Board. This determination was made by the local Institutional Review Board at the University of Arkansas for Medical Science (Little Rock, AR).

## Electronic supplementary material

Confirmation of AD LCL subgroups

## References

[1] Matelski L, Van de Water J (2016). Risk factors in autism: Thinking outside the brain. J Autoimmun.

[2] Thompson PA (2015). Environmental immune disruptors, inflammation and cancer risk. Carcinogenesis.

[3] Pessah IN (2008). Immunologic and neurodevelopmental susceptibilities of autism. Neurotoxicology.

[4] Hodjat M, Rezvanfar MA, Abdollahi M (2015). A systematic review on the role of environmental toxicants in stem cells aging. Food Chem Toxicol.

[5] Jose C, Melser S, Benard G, Rossignol R (2013). Mitoplasticity: adaptation biology of the mitochondrion to the cellular redox state in physiology and carcinogenesis. Antioxid Redox Signal.

[6] Rossignol DA, Genuis SJ, Frye RE (2014). Environmental toxicants and autism spectrum disorders: a systematic review. Transl Psychiatry.

[7] Wallace DC, Fan W (2010). Energetics, epigenetics, mitochondrial genetics. Mitochondrion.

[8] Frye RE, Rossignol DA (2011). Mitochondrial dysfunction can connect the diverse medical symptoms associated with autism spectrum disorders. Pediatr Res.

[9] Rossignol DA, Frye RE (2012). Mitochondrial dysfunction in autism spectrum disorders: a systematic review and meta-analysis. Mol Psychiatry.

[10] Frye, R. E. & Rossignol, D. Mitochondrial physiology and autism spectrum disorder. *OA Autism* **1** (2013).

[11] Wallace DC (2005). The mitochondrial genome in human adaptive radiation and disease: on the road to therapeutics and performance enhancement. Gene.

[12] Shaughnessy DT (2014). Mitochondria, energetics, epigenetics, and cellular responses to stress. Environ Health Perspect.

[13] Khusnutdinova E (2008). A mitochondrial etiology of neurodegenerative diseases: evidence from Parkinson’s disease. Ann N Y Acad Sci.

[14] Blossom SJ (2013). Metabolic changes and DNA hypomethylation in cerebellum are associated with behavioral alterations in mice exposed to trichloroethylene postnatally. Toxicol Appl Pharmacol.

[15] Bale AS, Barone S, Scott CS, Cooper GS (2011). A review of potential neurotoxic mechanisms among three chlorinated organic solvents. Toxicol Appl Pharmacol.

[16] Gash DM (2008). Trichloroethylene: Parkinsonism and complex 1 mitochondrial neurotoxicity. Ann Neurol.

[17] Hall GM, Kirtland SJ, Baum H (1973). The inhibition of mitochondrial respiration by inhalational anaesthetic agents. Br J Anaesth.

[18] Sauerbeck A, Hunter R, Bing G, Sullivan PG (2012). Traumatic brain injury and trichloroethylene exposure interact and produce functional, histological, and mitochondrial deficits. Exp Neurol.

[19] Martinez TN, Greenamyre JT (2012). Toxin models of mitochondrial dysfunction in Parkinson’s disease. Antioxid Redox Signal.

[20] Blossom SJ, Melnyk S, Cooney CA, Gilbert KM, James SJ (2012). Postnatal exposure to trichloroethylene alters glutathione redox homeostasis, methylation potential, and neurotrophin expression in the mouse hippocampus. Neurotoxicology.

[21] Blossom SJ, Melnyk SB, Li M, Wessinger WD, Cooney CA (2017). Inflammatory and oxidative stress-related effects associated with neurotoxicity are maintained after exclusively prenatal trichloroethylene exposure. Neurotoxicology.

[22] Blossom SJ, Gilbert KM (2006). Exposure to a metabolite of the environmental toxicant, trichloroethylene, attenuates CD4+ T cell activation-induced cell death by metalloproteinase-dependent FasL shedding. Toxicological sciences: an official journal of the Society of Toxicology.

[23] Gilbert KM, Whitlow AB, Pumford NR (2004). Environmental contaminant and disinfection by-product trichloroacetaldehyde stimulates T cells *in vitro*. International immunopharmacology.

[24] Blossom SJ, Pumford NR, Gilbert KM (2004). Activation and attenuation of apoptosis of CD4+ T cells following *in vivo* exposure to two common environmental toxicants, trichloroacetaldehyde hydrate and trichloroacetic acid. J Autoimmun.

[25] Blossom SJ, Doss JC, Gilbert KM (2007). Chronic exposure to a trichloroethylene metabolite in autoimmune-prone MRL+/+ mice promotes immune modulation and alopecia. Toxicological sciences: an official journal of the Society of Toxicology.

[26] Kang C, Li J,L (2012). Role of PGC-1alpha signaling in skeletal muscle health and disease. Ann N Y Acad Sci.

[27] Yoo HS (2015). Comparative analysis of the relationship between trichloroethylene metabolism and tissue-specific toxicity among inbred mouse strains: kidney effects. J Toxicol Environ Health A.

[28] Yoo HS (2015). Comparative analysis of the relationship between trichloroethylene metabolism and tissue-specific toxicity among inbred mouse strains: liver effects. J Toxicol Environ Health A.

[29] Jiang Y, Chen J, Tong J, Chen T (2014). Trichloroethylene-induced gene expression and DNA methylation changes in B6C3F1 mouse liver. PLoS One.

[30] Zaheer F, Slevin JT (2011). Trichloroethylene and Parkinson disease. Neurol Clin.

[31] Guehl D (1999). Trichloroethylene and parkinsonism: a human and experimental observation. Eur J Neurol.

[32] Kalkbrenner AE, Schmidt RJ, Penlesky AC (2014). Environmental chemical exposures and autism spectrum disorders: a review of the epidemiological evidence. Curr Probl Pediatr Adolesc Health Care.

[33] Zablotsky, B., Black, L. I., Maenner, M. J., Schieve, L. A. & Blumberg, S. J. Estimated Prevalence of Autism and Other Developmental Disabilities Following Questionnaire Changes in the 2014 National Health Interview Survey. *Natl Health Stat Report*, 1–20 (2015). 26632847

[34] Hallmayer J (2011). Genetic heritability and shared environmental factors among twin pairs with autism. Archives of general psychiatry.

[35] Sandin S (2014). The familial risk of autism. JAMA.

[36] Windham GC, Zhang L, Gunier R, Croen LA, Grether JK (2006). Autism spectrum disorders in relation to distribution of hazardous air pollutants in the san francisco bay area. Environ Health Perspect.

[37] James SJ (2006). Metabolic endophenotype and related genotypes are associated with oxidative stress in children with autism. Am J Med Genet B Neuropsychiatr Genet.

[38] Evangeliou A (2003). Application of a ketogenic diet in children with autistic behavior: pilot study. Journal of child neurology.

[39] Rose S (2012). Evidence of oxidative damage and inflammation associated with low glutathione redox status in the autism brain. Transl Psychiatry.

[40] Rossignol DA, Frye RE (2012). A review of research trends in physiological abnormalities in autism spectrum disorders: immune dysregulation, inflammation, oxidative stress, mitochondrial dysfunction and environmental toxicant exposures. Mol Psychiatry.

[41] Rose S (2014). Oxidative stress induces mitochondrial dysfunction in a subset of autistic lymphoblastoid cell lines. Transl Psychiatry.

[42] Rose S (2014). Oxidative stress induces mitochondrial dysfunction in a subset of autism lymphoblastoid cell lines in a well-matched case control cohort. PLoS One.

[43] Napoli E, Wong S, Hertz-Picciotto I, Giulivi C (2014). Deficits in bioenergetics and impaired immune response in granulocytes from children with autism. Pediatrics.

[44] Giulivi C (2010). Mitochondrial dysfunction in autism. JAMA.

[45] Rose S (2017). Mitochondrial and redox abnormalities in autism lymphoblastoid cells: a sibling control study. FASEB journal: official publication of the Federation of American Societies for Experimental Biology.

[46] Lu F (2000). Oxidative damage to mitochondrial DNA and activity of mitochondrial enzymes in chronic active lesions of multiple sclerosis. Journal of the neurological sciences.

[47] Mahad DJ (2009). Mitochondrial changes within axons in multiple sclerosis. Brain: a journal of neurology.

[48] Ekstrand MI (2007). Progressive parkinsonism in mice with respiratory-chain-deficient dopamine neurons. Proc Natl Acad Sci USA.

[49] James SJ (2009). Cellular and mitochondrial glutathione redox imbalance in lymphoblastoid cells derived from children with autism. FASEB journal: official publication of the Federation of American Societies for Experimental Biology.

[50] Wang G, Wang J, Ma H, Ansari GA, Khan MF (2013). N-Acetylcysteine protects against trichloroethene-mediated autoimmunity by attenuating oxidative stress. Toxicol Appl Pharmacol.

[51] Li LX, Skorpen F, Egeberg K, Jorgensen IH, Grill V (2001). Uncoupling protein-2 participates in cellular defense against oxidative stress in clonal beta-cells. Biochemical and biophysical research communications.

[52] Giardina TM, Steer JH, Lo SZ, Joyce DA (2008). Uncoupling protein-2 accumulates rapidly in the inner mitochondrial membrane during mitochondrial reactive oxygen stress in macrophages. Biochimica et biophysica acta.

[53] Fowler BA, Woods JS (1977). Ultrastructural and biochemical changes in renal mitochondria during chronic oral methyl mercury exposure: the relationship to renal function. Experimental and molecular pathology.

[54] Shenker BJ, Guo TL (1999). O, I. & Shapiro, I. M. Induction of apoptosis in human T-cells by methyl mercury: temporal relationship between mitochondrial dysfunction and loss of reductive reserve. Toxicol Appl Pharmacol.

[55] Goyer RA (1997). Toxic and essential metal interactions. Annual review of nutrition.

[56] Pourahmad J, Mihajlovic A, O’Brien PJ (2001). Hepatocyte lysis induced by environmental metal toxins may involve apoptotic death signals initiated by mitochondrial injury. Advances in experimental medicine and biology.

[57] Hiura, T. S. *et al*. The role of a mitochondrial pathway in the induction of apoptosis by chemicals extracted from diesel exhaust particles. *Journal of immunology* **165**, 2703–2711, doi:ji_v165n5p2703 [pii] (2000). 10.4049/jimmunol.165.5.2703 10946301

[58] Wong PW, Garcia EF, Pessah IN (2001). ortho-substituted PCB95 alters intracellular calcium signaling and causes cellular acidification in PC12 cells by an immunophilin-dependent mechanism. Journal of neurochemistry.

[59] Sherer TB (2007). Mechanism of toxicity of pesticides acting at complex I: relevance to environmental etiologies of Parkinson’s disease. Journal of neurochemistry.

[60] Yamano T, Morita S (1995). Effects of pesticides on isolated rat hepatocytes, mitochondria, and microsomes II. Archives of environmental contamination and toxicology.

[61] Samavati L, Lee I, Mathes I, Lottspeich F, Huttemann M (2008). Tumor necrosis factor alpha inhibits oxidative phosphorylation through tyrosine phosphorylation at subunit I of cytochrome c oxidase. The Journal of biological chemistry.

[62] Vempati UD (2007). Role of cytochrome C in apoptosis: increased sensitivity to tumor necrosis factor alpha is associated with respiratory defects but not with lack of cytochrome C release. Molecular and cellular biology.

[63] Suematsu N (2003). Oxidative stress mediates tumor necrosis factor-alpha-induced mitochondrial DNA damage and dysfunction in cardiac myocytes. Circulation.

[64] Vali S (2007). Integrating glutathione metabolism and mitochondrial dysfunction with implications for Parkinson’s disease: a dynamic model. Neuroscience.

[65] Fernandez-Checa JC (1997). GSH transport in mitochondria: defense against TNF-induced oxidative stress and alcohol-induced defect. The American journal of physiology.

[66] Graf WD (2000). Autism Associated With the Mitochondrial DNA G8363A Transfer RNA^Lys^ Mutation. Journal of child neurology.

[67] Frye RE, Naviaux RK (2011). Autistic disorder with complex IV overactivity: A new mitochondrial syndrome. Journal of Pediatric Neurology.

[68] Frye RE, Melnyk S, Macfabe DF (2013). Unique acyl-carnitine profiles are potential biomarkers for acquired mitochondrial disease in autism spectrum disorder. Transl Psychiatry.

[69] Palmieri L (2010). Altered calcium homeostasis in autism-spectrum disorders: evidence from biochemical and genetic studies of the mitochondrial aspartate/glutamate carrier AGC1. Mol Psychiatry.

[70] Anitha A (2012). Brain region-specific altered expression and association of mitochondria-related genes in autism. Molecular autism.

[71] Nie D (2015). The Stress-Induced Atf3-Gelsolin Cascade Underlies Dendritic Spine Deficits in Neuronal Models of Tuberous Sclerosis Complex. J Neurosci.

[72] Di Nardo A (2014). Neuronal Tsc1/2 complex controls autophagy through AMPK-dependent regulation of ULK1. Hum Mol Genet.

[73] Bartley AF (2015). Interneuron Transcriptional Dysregulation Causes Frequency-Dependent Alterations in the Balance of Inhibition and Excitation in Hippocampus. J Neurosci.

[74] Oh JH (2013). Genotype instability during long-term subculture of lymphoblastoid cell lines. Journal of human genetics.

[75] Nickles D (2012). In depth comparison of an individual’s DNA and its lymphoblastoid cell line using whole genome sequencing. BMC genomics.

[76] Lambert AJ, Brand MD (2004). Superoxide production by NADH:ubiquinone oxidoreductase (complex I) depends on the pH gradient across the mitochondrial inner membrane. The Biochemical journal.

[77] Nicholls DG (2009). Spare respiratory capacity, oxidative stress and excitotoxicity. Biochemical Society transactions.

[78] Yadava N, Nicholls DG (2007). Spare respiratory capacity rather than oxidative stress regulates glutamate excitotoxicity after partial respiratory inhibition of mitochondrial complex I with rotenone. J Neurosci.

[79] Sansbury BE, Jones SP, Riggs DW, Darley-Usmar VM, Hill BG (2011). Bioenergetic function in cardiovascular cells: the importance of the reserve capacity and its biological regulation. Chemico-biological interactions.

[80] Desler C (2012). Is There a Link between Mitochondrial Reserve Respiratory Capacity and Aging?. Journal of aging research.

[81] Hill BG, Dranka BP, Zou L, Chatham JC, Darley-Usmar VM (2009). Importance of the bioenergetic reserve capacity in response to cardiomyocyte stress induced by 4-hydroxynonenal. The Biochemical journal.

[82] Dranka BP, Hill BG, Darley-Usmar VM (2010). Mitochondrial reserve capacity in endothelial cells: The impact of nitric oxide and reactive oxygen species. Free radical biology & medicine.

[83] Choi SW, Gerencser AA, Nicholls DG (2009). Bioenergetic analysis of isolated cerebrocortical nerve terminals on a microgram scale: spare respiratory capacity and stochastic mitochondrial failure. Journal of neurochemistry.

[84] Coopman VA, Cordonnier JA, De Letter EA, Piette MH (2003). Tissue distribution of trichloroethylene in a case of accidental acute intoxication by inhalation. Forensic Sci Int.

[85] Beland FA (1999). NTP technical report on the toxicity and metabolism studies of chloral hydrate (CAS No. 302-17-0). Administered by gavage to F344/N rats and B6C3F1 mice. Toxic Rep Ser.

[86] Laird NM, Ware JH (1982). Random-effects models for longitudinal data. Biometrics.

