# Supplementary material for: Oxidative Stress Challenge Uncovers Trichloroacetaldehyde Hydrate-Induced Mitoplasticity in Autistic and Control Lymphoblastoid Cell Lines
Source: Sci Rep. 2017 Jun 30;7:4478. doi: 10.1038/s41598-017-04821-3 (PMC5493637; doi:10.1038/s41598-017-04821-3)
Supplement: Supplementary file 1 — Confirmation of AD LCL subgroups [file 41598_2017_4821_MOESM1_ESM.pdf]

***Scientific Reports*, Submitted**

Research Article

Oxidative Stress Challenge Uncovers Trichloroacetaldehyde Hydrate-Induced Mitoplasticity in Autistic and Control Lymphoblastoid Cell Lines

Running Title: Mitoplasticity and Oxidative Stress.

[1]Richard Eugene Frye, M.D., Ph.D., [1]Shannon Rose, Ph.D., [1]Rebecca Wynne, M.S.,

[1]Sirish C. Bennuri, B.S, [1]Sarah Blossom, Ph.D., [1]Kathleen M. Gilbert, Ph.D.,

[2]Lynne Heilbrun, M.P.H and [2]Raymond F. Palmer, Ph.D.

[1]Arkansas Children's Research Institute, Little Rock, AR USA and Department of Pediatrics, University of Arkansas for Medical Sciences, Little Rock, AR USA; [2]Department of Family and Community Medicine, University of Texas Health Science Center, San Antonio, TX

Confirmation of AD LCL subgroups

To verify that the AD subgroups (AD-A and AD-N) showed the same pattern of respiratory abnormalities as we previously reported, we examined the respiratory parameters with DMNQ challenge in LCLs not exposed to TCAH (Figure S1). We only perform post hoc tests that include the group factor, not the DMNQ factor alone.

<u>ATP-Linked Respiration</u>

ATP-Linked Respiration (Figure S1A) significantly changed across Groups [$F(2,176)=31.93$, $p<0.001$] and DMNQ [$F(2,18)=8.45$, $p<0.005$]. As expected AD-A LCLs had significantly higher ATP-Linked Respiration compared to Control/AD-N LCLs [$t(176)=7.58$, $p<0.0001$]. This is consistent with previous findings on ATP-Linked Respiration in AD-A LCLs.

<u>Proton Leak Respiration</u>

Proton Leak Respiration (Figure S1B) significantly changed across Groups [$F(2,176)=13.32$, $p<0.0001$] and DMNQ [$F(2,18)=56.37$, $p<0.0001$] with a borderline significant Group by DMNQ interaction [$F(4,176)=2.08$, $p=0.09$]. As expected AD-A LCLs demonstrated significantly higher Proton Leak Respiration as compared to Control/AD-N LCLs [$t(176)=4.32$, $p<0.0001$]. Consistent with previous studies, the increase in Proton Leak Respiration with higher DMNQ concentrations was significantly greater in the AD-A LCLs as compared to the Control/AD-N LCLs. Indeed, Proton Leak Respiration was not different between AD-A LCLs and Control/AD-N LCLs at baseline (0uM DMNQ) but became significantly higher in the AD-A LCLs at 5uM [$t(176)=2.67$, $p<0.01$] and 10uM [$t(176)=4.20$,$p<0.0001$] DMNQ.

<u>Maximal Respiratory Capacity</u>

Maximal Respiration (Figure S1C) significantly changed across Groups [$F(2,176)=8.21$, $p<0.0005$] and DMNQ [$F(2,18)=18.84$, $p<0.0001$]. Consistent with previous studies, Maximal

Respiratory Capacity was significantly higher in AD-A LCLs as compared to Control/AD-N LCLs [t(176)=3.05, p<0.005].

Reserve Capacity

Reserve Capacity (Figure S1D) significantly changed across Groups [F(2,176)=3.06, p=0.05] and DMNQ [F(2,18)=30.13, p<0.0001] with a significant Group by DMNQ interaction [F(4,176)=5.23, p=0.0005]. Overall Reserve Capacity was higher in AD-A LCLs [t(176)=2.22, p=0.03] as compared to the AD-N/Control group. Consistent with our previous studies, Reserve Capacity decreased quicker as DMNQ increased in the AD-A LCLs. Indeed, AD-A LCLs demonstrated a significantly higher Reserve Capacity at baseline (i.e., 0uM) [t(176)=2.42, p=0.02] and significantly lower Reserve Capacity at higher DMNQ concentrations [5uM:t(176)=3.07, p<0.005; 10uM:t(176)=2.99, p<0.005] as compared to Control/AD-N LCLs.

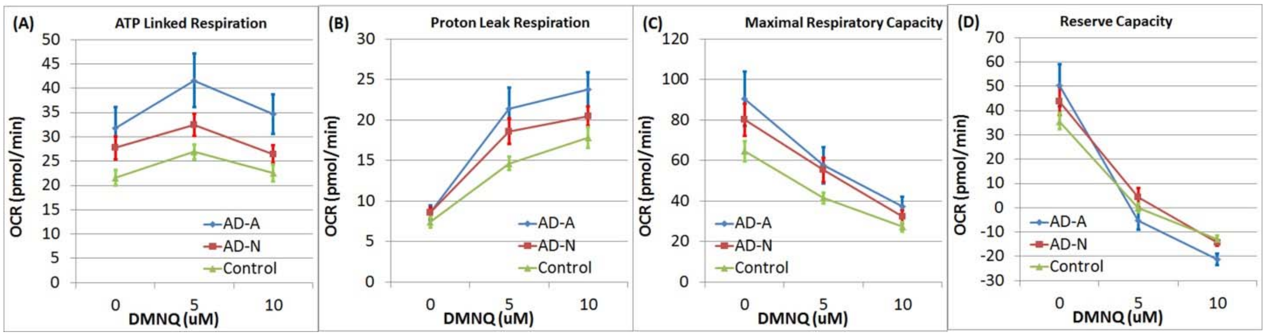

Figure S1. **Mitochondrial Respiratory Parameters Compared Across Lymphoblastoid Cell Line (LCL) Groups with No Trichloroacetaldehyde hydrate Exposure. (**A) **ATP-linked Respiration,** (B) **Proton Leak Respiration,** (C) **Maximal Respiratory Capacity** and (D) **Reserve Capacity** are significantly higher in the AD-A LCL group as compared to the Control/AD-N LCL groups. (D) In addition, **Reserve Capacity** demonstrated a greater decline with increasing 2,3-dimethoxy-1,4-napthoquinone concentrations.
